# Extreme UV sensitivity of native *Metarhizium* spp. as potential biocontrol agent for False Codling Moth (*Thaumatotibia leucotreta* Meyrick) on chili pepper in Ghana

**DOI:** 10.3389/ffunb.2025.1660692

**Published:** 2025-08-25

**Authors:** Patricia Akua Sitsofe Nyahe, Vincent Yao Eziah, Laith Khalil Tawfeeq Al-Ani, Monica Akumyoungta, Candice Anne Coombes, Drauzio Eduardo Naretto Rangel, Alene Alder-Rangel, Dalia Sukmawati, Owusu Fordjour Aidoo, Mavis Agyeiwaa Acheampong

**Affiliations:** ^1^ Department of Crop Science, University of Ghana, Accra, Ghana; ^2^ School of Biological Science, Universiti Sains Malaysia, George, Pulau Pinang, Malaysia; ^3^ Centre for Biological Control (CBC), Department of Zoology and Entomology, Rhodes University, Makhanda, South Africa; ^4^ Inbioter – Institute of Biotechnology Rangel, Itatiba, Brazil; ^5^ Alder’s English Services, São José dos Campos, Brazil; ^6^ Department of Biology, Faculty of Mathematics and Natural Sciences, Universitas Negeri Jakarta, Jakarta, Indonesia; ^7^ Department of Entomology, Washington State University, Pullman, WA, United States

**Keywords:** chili pepper, entomopathogenic fungi, False Codling Moth, *Metarhizium* spp., simulated solar radiation, UV tolerance, virulence

## Abstract

Chili pepper exports from Ghana are subject to stringent chemical residue regulations in key export destinations. Consequently, microbial biopesticides are urgently needed to complement current nonchemical control options for key pests of chili pepper, particularly the phytosanitary insect, False Codling Moth (FCM). Thus, the search for native entomopathogenic fungi in Ghanaian farms was initiated in 2023. Seven *Metarhizium* isolates (UGSUHCI, UGJKCS9, UGJKCS10, UGAFMF8, UGAFM F12, UGNAKC1 and UGKAP1), obtained from agricultural soils in Ghana, showed high virulence against the soil-dwelling stages of FCM under laboratory conditions. To facilitate the selection of these virulent isolates for development into a mycoinsecticide for FCM, the UV sensitivity and virulence following UV exposure were investigated for all seven isolates in this study. All isolates exhibited extreme susceptibility to UV radiation in comparison to similar research. Exposure to simulated full-spectrum solar radiation at 0.6 W/m^2^ for 30 min reduced relative conidial germination by 28–40% 48 h following exposure, while 60 min exposure killed all isolates. High insect mortalities were recorded for four isolates, regardless of UV radiation. The findings suggest that an effective UV-protectant formulation could be required for success in the field against fruit and foliar pests of chili pepper, including those of FCM.

## 1 Introduction

Chili pepper (*Capsicum annuum* L.) is a key ingredient in daily diets of Ghanaians, making it the fourth most planted crop in the country, with a current annual average of 140,000 MT (GSS, 2014; MoFa-IFPRI, 2020). Besides the high local demand, this crop is one of Ghana’s top export vegetables to the lucrative European Union (EU) market, which increases annually, especially for the Legon 18 variety, known for its exceptional taste and long shelf life (GEPA, 2021). Chili pepper is therefore cultivated year-round in all 16 regions in Ghana, with the Volta, Eastern and Northern regions of Ghana being the highest producers (GEPA, 2018).

False Codling Moth (FCM) (*Thaumatotibia leucotreta* Meyrick, Lepidoptera: Tortricidae) is the major impediment to export, as this pest is strictly regulated as a phytosanitary organism in the EU (EPPO, 2013), Ghana’s main chili pepper export market. Local production is significantly constrained by this pest, whose larvae develop within fruits, resulting in immature fruit ripening, dropping of fruits and fruit decay, which lead to yield losses (Adom et al., 2023; Adom et al., 2024). The frequent interceptions of this pest resulted in the EU banning the import of chili peppers from Ghana between 2015 and 2017. This, together with the prohibition of two other vegetables (gourd and eggplant), cost the nation an estimated export revenue loss of USD 30 million (EUROPHYT, 2014; Fening et al., 2017). This has resulted in a drastic reduction in chili pepper exports in Ghana, as demonstrated by decreasing export volumes and values between 2010 to 2014 (984,374–1,079,882 kg with corresponding earnings of USD 350,442–1,184,964 (GEPA, 2021) compared to 2018 to 2021 (USD 351,000–87,000) (GEPA, 2018; GIRSAL, 2025).

The use of synthetic pesticides, which remains the main control method for FCM in Ghana, has not been sufficient, partly due to the narrow window for controlling the inconspicuous eggs and neonates of FCM on fruits. Apart from unsatisfactory control with conventional pesticides, they are also stringently regulated by the key export markets and have adverse effects on the environment, non-target organisms, and human health (Cech et al., 2023; Wan et al., 2025). Therefore, effective and sustainable nonchemical control options are needed to control this pest in Ghana. Although commercially available *Bacillus thuringiensis* (Bt)-based products in Ghana have been proven useful in the control of the above-ground life stages of FCM (Adom et al., 2023), additional control agents for the non-feeding soil-dwelling life stages of the pest are needed, leading to the search for native entomopathogenic fungi (EPF).

Seven native EPF obtained from agricultural soils in Ghana (**Table 1**) have shown promise as control agents for the soil-dwelling stages of FCM under laboratory conditions, inducing over 80% pupal mortality of FCM (Acheampong M.A. Unpublished data). However, abiotic environmental constraints, particularly ultraviolet (UV) radiation, are well documented to be among the key efficacy impeding factors of EPF in the field and must be factored into the isolate selection process (Braga et al., 2001a; Posadas et al., 2012; Kaiser et al., 2019; Acheampong et al., 2020a).

**Table 1:** Origin and molecular analyses of seven soil-derived Ghanaian *Metarhizium* spp. used in the study.

Isolate	Geographical origin	Farm type^1^	Date of isolation	GenBank accession number^2^
UGJKCS9	5.265305 N 1.340426 W	Cocoa	16/02/2024	*
UGJKCS10	5.265125 N 1.339959 W	Cocoa	16/02/2024	PQ778821
UGSUHC1	6.05856 N 0.40275 W	Cocoa	03/12/2023	PQ800172- PQ800173
UGNAKC1	6.076370 N 0.374230 W	Cocoa	03/12/2023	PQ781266
UGKAP1	6.5885008 N 0.8282585 W	Chili pepper	07/12/2023	PQ805341-PQ805342
UGAFMF8	5.7824166 N 0.608745 E	Maize	05/05/2024	PQ778958
UGAFM12	5.783545 N 0.610471 E	Maize	05/05/2024	*

^1^All isolates were baited with *Galleria mellonella*.

^2^Molecular analyses based on the ITS1 and ITS4 regions.

*Metarhizium anisopliae sensu lato.

Among the UV radiation emitted from sunlight, UV-B is the most damaging to entomopathogens (Rangel and Roberts, 2018), inhibiting replication and inducing mutations and cellular mortality (Rangel et al., 2006; Nascimento et al., 2010; Wang et al., 2019), whereas UV-A exposure stimulates the generation of detrimental radicals, which deactivate propagules (Rangel et al., 2006). Nevertheless, the susceptibility of EPF to UV radiation is isolate and species dependent (Fernandes et al., 2007; Posadas et al., 2012; Fernandes et al., 2015; Acheampong et al., 2020a; Licona-Juárez et al., 2023; Rangel et al., 2023). Consequently, identifying UV-resilient EPF strains and formulating them with appropriate UV protectants can enhance their persistence in UV-exposed environments, resulting in greater efficacy against pests (Posadas et al., 2012; Fernandes et al., 2015; Kaiser et al., 2019). While UV-radiation may not be the most inimical abiotic factor for the EPF when applied to control the soil-dwelling stages, the biopesticide product developed will ultimately be used to also target above-ground stages of FCM and other foliar pests of pepper. Thus, this research investigated the UV tolerance of all seven promising native EPF isolates to select the most suitable for the chili pepper environment.

## 2 Materials and methods

### 2.1 Source of insects, fungal isolates and culture conditions

All seven native *Metarhizium* isolates were obtained from the Entomopathology Laboratory of the African Regional Postgraduate Programme in Insect Science (ARPPIS), University of Ghana, where conidia had been stored on Sabouraud dextrose agar (SDA) slants at 4°C. These EPF were isolated from soils from chili pepper, maize and cocoa farms in the Central, Eastern and Greater Accra regions of Ghana using *Galleria mellonella* (Lepidoptera: Pyralidae) (Goble et al., 2010) (**Table 1**). All isolates were passed through FCM fifth instar larvae once before use, following the protocol of Acheampong et al. (2020a). Cadaver cultures were maintained on SDA medium supplemented with 50 mg/L chloramphenicol (SDAC) and kept at 4°C, serving as stock cultures for the UV assays. The FCM final (fifth) instar larvae used in this study were obtained from the Centre for Biological Control, Rhodes University, Makhanda, South Africa, where a continuous rearing culture of this insect is held using an artificial larval diet (Moore et al., 2014).

### 2.2 Simulated solar radiation device

Irradiation tests were carried out in a Q-SUN^®^ Xe-3-HC (Q-Lab Corporation, Westlake, OH, USA) solar radiation simulator. The Q-SUN^®^ reproduces full-spectrum solar radiation from 295 to 780 nm, using three 1800 W Xenon arc lamps and a Daylight-Q filter, which excludes radiation below 295 nm (Dias et al., 2018). The uniform spectral distribution on irradiation surfaces produced by Q-SUN^®^ lamps, enhanced by mirrored walls, facilitates reproducible results. The strong correlation of these lamps to sunlight is well established (https://www.q-lab.com) and recently validated in assessing UV tolerances of EPF (Luo et al., 2017; Dias et al., 2018; Acheampong et al., 2020a).

The Q-SUN^®^ was calibrated to 0.6 W/m^2^ irradiance at a temperature of 23.2 ± 0.66°C and relative humidity (RH) of 63 ± 10.4% RH. This irradiance set point is less than the average annual daily solar radiation in Ghana of 4–6 kWh/m^2^ (Edjekumhene and Brew-Hammond, 2001; Aboagye et al., 2021). The Quaite-weighted (biologically effective UV dosage capable of DNA damage in some fungi including EPF) (Quaite et al., 1992; Braga et al., 2001b) irradiance in the Q-SUN^®^ at 0.6 W/m^2^ is 1335 mW/m^2^, which also approximates noon irradiance during summer in Sao Jose dos Campos (Luo et al., 2017; Dias et al., 2018), South-Eastern Brazil (Dias et al., 2018), with a similar climate to Southern Ghana. Thus, the irradiance used could be lower than the Quaite-weighted noon irradiance in Ghana’s chili pepper producing regions. In a previous study, 3 h of exposure to simulated full-spectrum solar radiation in the Q-SUN^®^ at 0.6 W/m^2^ killed over 90% of conidia of 11 tested EPF isolates (ARSEF collections of *Aschersonia aleyrodis* (Brazil*)*; *Beauveria bassiana*, *Isaria fumosorosea*, *Metarhizium robertsii* and *Tolypocladium inflatum* from USA; *Lecanicillium aphanocladii* from Brazil; *Marianneae pruinose* from China*, M. anisopliae* s.l. from Mexico, *M. brunneum* from New Zealand, *Simplicillium lanosoniveum* from French Guiana, and *T. cylindrosporum* from Nepal) after 48 h of exposure, whilst germination in their non-irradiated controls exceeded 95% after 24 h (Dias et al., 2018). This result demonstrated that the selected irradiance set point and a maximum exposure duration of 2 h were appropriate for determining the tolerance of these EPF.

### 2.3 Effect of simulated solar radiation on conidial germination of the isolates

Conidial viability of suspensions to be applied was determined by plating aliquots of conidial suspension [50 mL of 10^5^ conidia/mL from 14-d-old cultures suspended in Tween 20 (0.01% v/v)] onto SDA medium in three replicate Petri plates (Polystyrene, 60 × 15 mm). Plates were incubated at 26 ± 1°C for 12 h, after which the germinated and non-germinated conidia per plate, out of 300 conidia, were evaluated. Germination was assessed at 400× magnification; conidia were considered germinated when the germ tube was longer than the diameter of the conidium (Rangel et al., 2005).

Only three fungal isolates and their control were irradiated on each occasion due to the limited space in the Q-SUN^®^. Stock cultures of each isolate were sub-cultured on SDAC and incubated for 12–15 days at 27°C, 60% RH, on a 12 h photoperiod. Conidia produced were then harvested from colonies, suspended in sterile distilled water supplemented with 0.01% Tween 20, and adjusted to 1 × 10^5^ conidia/mL. For each isolate, a 50 µL suspension was spread across a 60 mm SDA Petri plate in four replicates for four exposure periods, including controls. Aluminum foil was used to wrap control plates to block UV radiation (Braga et al., 2001a; Acheampong et al., 2020a). The Petri plates were exposed to simulated full-spectrum solar radiation in the Q-SUN^®^ within 30 min after inoculation at 0.6 W/m^2^ for 15, 30, 60 and 120 min, corresponding to total doses of 1.20, 2.40, 4.81 and 9.61 kJ/m^2^, respectively. Following irradiation, plates were incubated in the dark at 20 ± 1°C. The number of germinated (conidia with germ tubes) and non-germinated conidia per plate, out of 300 conidia, was assessed 24 h and 48 h following irradiation, using an optical microscope. The entire experiment was repeated three times for each isolate, using fresh conidial suspension.

### 2.4 Effect of simulated solar radiation on the virulence of fungal isolates

For each isolate, 1.5 mL conidial suspension (10^6^ conidia/mL suspended in 0.01% Tween 20) in a 10 mL sterile glass vial was exposed to the same dose and irradiance period as the UV tolerance assay. After exposure, the vial was vortexed for 1 min, and 0.1 mL of suspension was then pipetted and spread in a 60 mm sterile plastic Petri plates lined with sterile filter paper at four replicates. Ten FCM fifth instar larvae were immediately added to each Petri plate and incubated for 14 days at 25°C. Non-irradiated inoculated vials of each isolate (wrapped with aluminum foil to block UV radiation) and non-inoculated control insects (in sterile Petri plates lined with filter paper and treated with 0.1 mL of 0.01% Tween 20) served as controls. The number of dead and live insects was assessed daily for 14 days after treatment. Death due to mycosis was verified by surface sterilizing cadavers in 0.5% sodium hypochlorite (3.5% active ingredient), followed by 70% ethanol for 2 min each and kept in Petri plates lined with filter papers, and moistened with sterile water for seven days at 25°C. The entire experiment was repeated twice.

### 2.5 Statistical analyses

The percentage of germination of all isolates relative to control plates was calculated according to Acheampong et al. (2020a). The 60- and 120-min fungal exposure data for both UV and pathogenicity assays were excluded from the analysis as conidia of all isolates were killed at both periods. The relative germination data were analyzed using a generalized linear model (GLM) with gamma error distribution (link= ‘inverse’), which produced the best goodness of fit (lowest Akaike Information Criteria value) based on Likelihood Ratio Test (LRT) (Acheampong et al., 2020a). A three-way analysis of deviance (ANODEV) was applied to the model and contrasted using the ‘emmeans’ R package (Lenth, 2024), adjusted with Tukey’s HSD test (P ≤ 0.05). The cumulative FCM larval, pupal and adult mortality data at each exposure over the 14-day period were pooled and firstly fitted to a logistic regression in a GLM with binomial error distributions to determine an interaction effect. The mortality data for each exposure period were then subjected to logistic regression followed by pairwise comparison of treatment means using ‘emmeans’, adjusted with Tukey’s HSD test (P ≤ 0.05), where statistical differences were noted. All analyses were done in R version 4.4.2 (R Core Team, 2024).

## 3 Results

### 3.1 Effect of simulated solar radiation on conidial germination of the isolates

The three-way ANODEV showed that only fungal isolate (LRT, χ^2^ = 0.38, df = 6, P < 0.001) and the exposure period (LRT, χ^2^ = 1.67, df = 1, P < 0.001) significantly influenced conidial germination. The incubation period (LRT, χ^2^ = 0.01, df = 1, P = 0.182) and associated interactions [fungal isolate × incubation (LRT, χ^2^ = 0.01, df = 6, P = 0.967), incubation period × exposure period (LRT, χ^2^ = 0.00, df = 1, P = 0.586), fungal isolate × exposure period × incubation period (LRT, χ^2^ = 0.00, df = 6, P = 0.999)] were not significant. However, fungal isolate × exposure period (LRT, χ^2^ = 0.09, df = 6, P = 0.011) was significant.

**Figure 1:**
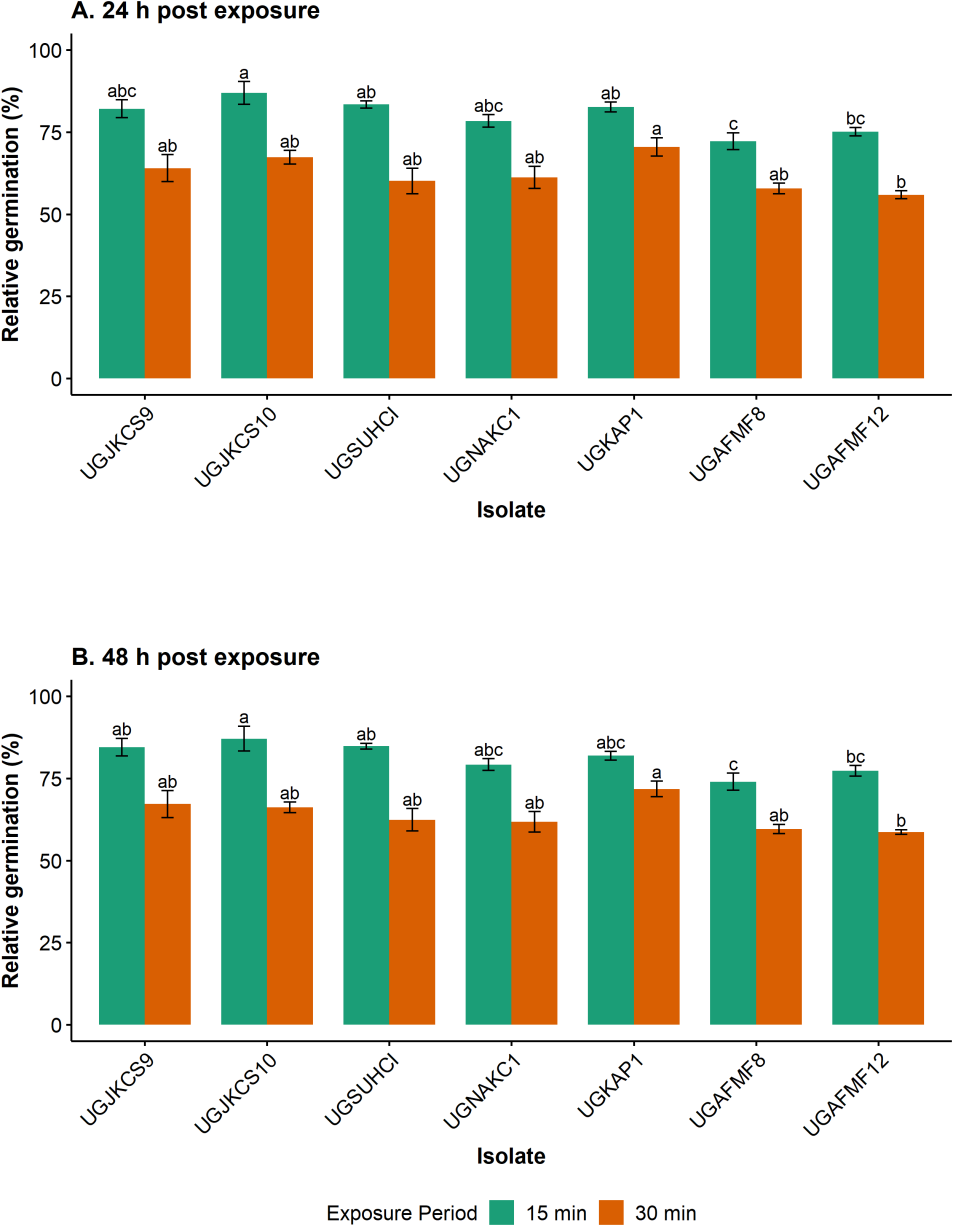
Relative percentage germination of seven *Metarhizium* isolates after exposure to simulated solar radiation (Xenon arc lamps from 295 to 780 nm at 0.6 W/m^2^, 28 ± 1°C and 46 ± 3.19% RH) for 0 (control), 15 (1.2 kJ/m^2^) and 30 (2.4 kJ/m^2^) min, and incubated for 24 **(A)** and 48 **(B)** h at 20 ± 1°C. Error bars are the standard errors of three independent experiments with a fresh batch of conidia. All statistical comparisons were done for each exposure period (but not between each isolate at the two exposure periods). Means within each exposure period with the same lowercase letters are not significantly different (‘emmeans’ adjusted with Tukey’s HSD test, P > 0.05).

Exposure to simulated solar radiation for 15 min (1.2 kJ/m^2^) had little impact on four fungal isolates, with their relative germination exceeding 82% after 24 h of incubation (**Figure 1A**). UGAFMF8 was the most susceptible isolate at this exposure time, with 72% relative germination 24 h following incubation.

Exposure for 30 min (2.4 kJ/m^2^) reduced conidial germination of isolates by 29–44% after 24 h of incubation. There were, however, indiscernible differences in susceptibility among six of these isolates at this exposure period. UGAFMF12 was the most susceptible isolate at this exposure period, only differing significantly from UGKAP1.

For both exposure periods, the incubation of isolates for a further 24 h increased germination only marginally. The relative germination of isolates ranged from 74–87% and 60–72% for the 15- and 30-min exposure periods, respectively, after 48 h of incubation (**Figure 1B**). Exposure to simulated solar radiation for 60 min (4.81 kJ/m^2^) and 120 min (9.61 kJ/m^2^) killed conidia of all tested isolates.

### 3.2 Effect of simulated solar radiation on the virulence of the isolates

Fungal isolate (LRT, χ^2^ = 675.88, df = 14, P < 0.001) and exposure period (LRT, χ^2^ = 9.80, df = 1, P = 0.002) significantly influenced pupal mortality. However, their interaction was not significant (LRT, χ^2^ = 17.82, df = 14, P = 0.215).

**Figure 2:**
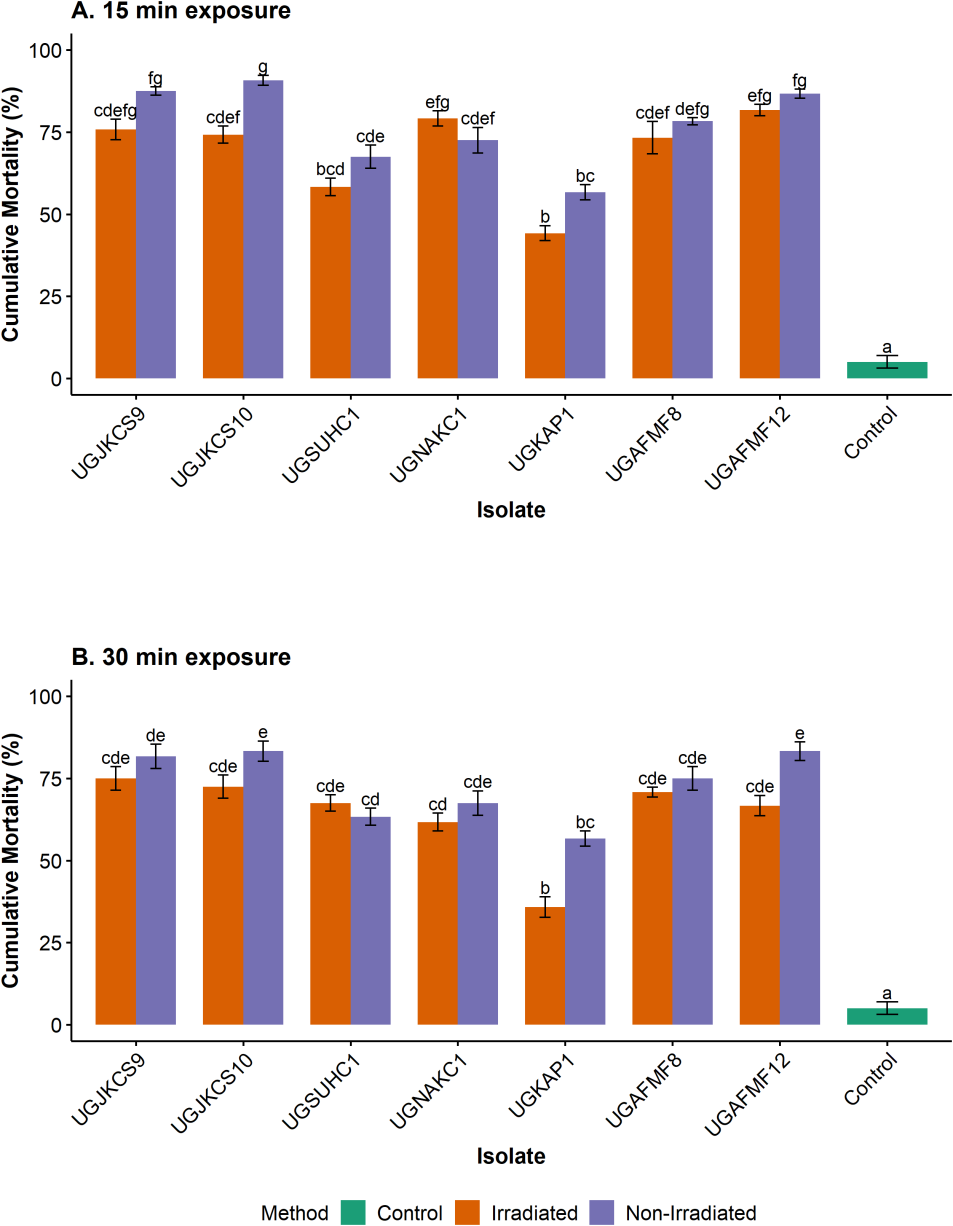
Cumulative percentage mortality of larvae, pupae and adults of *T. leucotreta* treated with *Metarhizium* isolates exposed to simulated solar radiation (Xenon arc lamps from 295 to 780 nm at 0.6 W/m^2^, 28 ± 1°C and 46 ± 3.19% RH) for 0 (control), 15 **(A)** and 30 **(B)** min and incubated for 14 days at 25°C. Error bars are the standard errors of three independent experiments with a fresh batch of conidia. Means within each exposure period with the same lowercase letters are not significantly different (‘emmeans’ adjusted with Tukey’s HSD test, P > 0.05).

For each isolate, insect mortality induced by irradiated conidia for 15 (1.2 kJ/m^2^) and 30 (2.4 kJ/m^2^) min was not significantly different from the non-irradiated control, with the exception of UGJKCS10, whose mortality in both treatments differed at the shortest exposure period (**Figure 2**). High pupal mortalities were recorded for four isolates (UGJKCS9, UGJKCS10, UGAFMF8 and UGAFMF12) regardless of UV radiation. Mortality induced by these isolates ranged from 73–82% and 67–75% at 15- and 30-min exposures, respectively. The mortality of the insects in the control, which was neither irradiated nor inoculated, remained at 5% and was significantly lower than all treatments in all bioassays (**Figure 2**).

## 4 Discussion

UV radiation is well established to be the most important abiotic environmental constraint to the efficacy of biopesticides in the field. However, the location of the targeted insect pest could help prioritize superior UV protectants in the formulation stage of biopesticide development. While UV radiation may not be the most important abiotic efficacy impeding factor for EPF applied to control the soil-dwelling stages (pre-pupating final larvae, pupae) of FCM in this research, other fruit and foliar pests of pepper, particularly thrips, aphids, whiteflies and fruit flies, are equally important and would be targeted. EPF have been used to successfully suppress thrips (Arthurs et al., 2013; Panyasiri et al., 2022), aphids (Mantzoukas et al., 2022) and whiteflies (Avery et al., 2020; Zulfitri et al., 2020) on chili pepper plants in other countries. Furthermore, the above-ground stages of the targeted pest of this research (adults, eggs and neonates) ought to be controlled, hence the need to factor UV resilience in the strain selection.

All indigenous isolates tested in this study exhibited extreme sensitivity to UV radiation, which generally aligns with other EPF-UV sensitivity studies (Braga et al., 2001a; Posadas et al., 2012; Kaiser et al., 2019; Acheampong et al., 2020a). Nonetheless, the total inactivation after only 1 h of exposure contradicts previous findings using the same simulated sunlight device and irradiance (Dias et al., 2018) and others where propagules were completely killed or had a > 50% reduction in viability only after 2–8 h of exposure using monochromatic (Braga et al., 2001a, Braga et al., 2002; Fernandes et al., 2007; Santos et al., 2011; Kaiser et al., 2019) and polychromatic (Alves et al., 1998; Leland and Behle, 2005; Luo et al., 2017) light sources, even at higher irradiances. It is also acknowledged that isolates inherent genetic variability, and geographical origin, in addition to methodological differences (formulation status, culture age, conidial densities and condition in storage and prior to irradiation, amongst others) in some of the aforementioned studies could account for the differences in UV sensitivities compared to the present study.

Exposure to simulated solar radiation for both 15 and 30 min had no impact on the pathogenicity of all seven EPF isolates investigated in this study. These findings corroborate those of Fernández-Bravo et al (2017); Fernández-Bravo et al, 2024) who reported a negative correlation between loss of viability and infection potential against Mediterranean fruit fly (*Ceratitis capitata*) of three *Beauveria bassiana* (EABb 10/225-Fi, EABb 09/20-Fi and EABb 09/28-Fil) and a *Metarhizium brunneum* (EAMa 01/58-Su) isolates, following exposure to UV-B radiation (1200 mW/m^2^) for 6 h. A similar insignificant effect of UV radiation (UV-A and UV-B) on mortality of FCM was reported with a *B. bassiana* and three *Metarhizium* spp. under laboratory conditions by Rossouw et al. (2023). Likewise, 8 h of UV-A exposure (31.514 mW/m^2^) of *Leptolegnia chapmanii* zoospores did not affect its *in vitro* virulence against yellow fever mosquito (*Aedes aegypti*) larvae (Páramo et al., 2015). However, Rossouw et al. (2023) recorded low persistence and mortalities in field trials with unformulated (aqueous conidial suspension) isolates and highlighted the need for an appropriate UV-protectant formulation to enhance field persistence and efficacy.

Although the most UV-tolerant isolate was elucidated at 30 min, isolates may need to persist for longer than this period to achieve success against insect pests in the phyllosphere environment of chili pepper in Ghana. However, the microclimate within the hypogeal environment of chili pepper could be conducive for infection of the soil-dwelling stages of FCM due to possible shade protection by the canopy of this plant, as reported in other crop systems. For instance, *Betabaculovirus cryleucotreta*, which is a virus of FCM, exhibited better persistence on the southern side of the trees in Hermitage Farm (33°32’02” S and 25°40’13” E) in the Sunday’s River Valley, Eastern Cape, South Africa, as opposed to the northern sides (which receives higher UV exposure) because of some protection afforded by the trees themselves (shade) (Mwanza, 2015). In recent years, EPF isolates occurring in the phyllosphere environment have been sought after due to their perceived greater tolerance to UV radiation and heat than isolates obtained from soils, with some evidence (Vidal et al., 1997; Bidochka et al., 2001; Braga et al., 2001b; Bidochka et al., 2002; Jaronski, 2010). However, contrary reports exist (Fargues et al., 1996, Fargues et al., 1997; Leland and Behle, 2005; Fernandes et al., 2007; Fernández-Bravo et al., 2016; Acheampong et al., 2020a; Acheampong et al, 2020b). This indicates that UV tolerance could depend more on the isolate than the geoclimatic origins or isolation habitats.

Given the high UV susceptibility of these isolates, a suitable formulation will need to be identified if they are to be used in a commercial setting. Greenyield Ltd (Pato Branco, Paraná, Brazil) has developed a novel adjuvant product, Green Turbo^®^, for the biopesticide industry. This product, which contains extracts of algae, plants and essential oils, provided excellent *in vitro* photoprotection of conidia of some entomopathogenic and mycoparasitic fungi (Acheampong, M. A. Unpublished data). The protection of propagules against UV radiation of this formulation, although yet to be established, has been attributed to the potential mycosporines and mycosporine-like amino acids (MAA) produced as secondary metabolites by the algae, coupled with the oils. The exact oils used in Green Turbo^®^ formulation are not known; however, several mineral and vegetable oils are well documented to be capable of protecting propagules of EPF against UV radiation (Leland and Behle, 2005; Posadas et al., 2012; Kaiser et al., 2019; Acheampong et al., 2020a) and are widely utilized in the biopesticide industry. Similarly, mycosporines and MAA are well-known organic sunscreens produced by algae and other organisms (Katoch et al., 2016; Chrapusta et al., 2017; Geraldes and Pinto, 2021; Punchakara et al., 2023) and are widely used in the pharmaceutical and cosmetic industries (Chrapusta et al., 2017; Rosic, 2019; Thiyagarasaiyar et al., 2020). Thus, the new UV-protectant adjuvant formulation, Green Turbo^®^, is currently being investigated for potential photoprotection of the EPF isolates studied. Nonetheless, the UV sensitivity findings in this study indicate that application of isolates at sunset could ensure persistence on foliage and fruits for infection of nocturnal stages of the main targeted insect pest of this research (FCM), provided attachment of conidia to penetration of the insect host occurs within 24 h as proposed (Jaronski, 2010) and observed in *Anastrepha fraterculus* (Wiedemann; Diptera: Tephritidae) (Bechara et al., 2011).

## 5 Conclusions

The manuscript highlights the potential of seven native *Metarhizium* spp. isolates from Ghana as biological control agents against FCM affecting chili pepper. The main perspective of the study is that, despite their promising virulence against the soil-dwelling stages of FCM, all isolates exhibited extreme sensitivity to UV radiation, with complete conidial inactivation occurring after just one hour of simulated solar exposure. However, the pathogenicity of the isolates remained unaffected at lower exposure durations (15–30 min), suggesting their short-term viability in field conditions. The study emphasizes the importance of developing UV-protectant formulations or applying the isolates during low UV periods (e.g., at sunset) to potentially enhance persistence and infection success against above-ground pests. The key limitation is the poor UV tolerance of the isolates, which significantly hinders their potential as stand-alone biopesticides under natural sunlight, thereby requiring further research on formulation and application strategies to ensure practical field application.

## Data Availability

The raw data supporting the conclusions of this article will be made available by the authors, without undue reservation.
